# The Outcome of Surgery versus Medical Management in the Treatment of Vesicoureteral Reflux

**DOI:** 10.1155/2008/437560

**Published:** 2008-07-21

**Authors:** Caleb P. Nelson

**Affiliations:** Department of Urology, Children's Hospital Boston, Harvard Medical School, Boston, MA 02115, USA

## Abstract

Evaluation of the relative merits of medical versus surgical management of vesicoureteral reflux (VUR) has been limited by the few prospective studies comparing these strategies. Among those trials that have been reported, the only consistent positive finding has been that incidence of febrile UTI is lower among children undergoing surgical treatment in comparison with medical treatment. Studies have not found significant differences in overall incidence of UTI, or in rates of new renal scarring or progression of existing scarring. It is likely that there is a subset of children with VUR who do benefit from aggressive treatment of their VUR, but we are not yet able to fully determine which children these are. It is hoped that future research will further clarify which treatments are useful in which children.

## 1. INTRODUCTION

Urinary tract infection (UTI) is one of the most common serious bacterial infections in children. Cumulative incidence is 1–2% among boys and 3–7% among girls, and between 70 000 to 180 000 of the annual US birth cohort will have a UTI by the age of 6 [1]. Roughly 30–50% of children with UTI are found to have vesicoureteral reflux (VUR, the retrograde flow of urine from the bladder into the ureter and/or kidney). Because VUR (particularly when coexistent with UTI) has been associated with increased risk of renal scarring, proteinuria, hypertension, eclampsia, and end-stage renal disease (ESRD) [2], children with UTI typically undergo diagnostic evaluation for and treatment of VUR.

In addition to its association with UTI, VUR is also a highly genetic condition, displaying an autosomal dominant transmission pattern, with variable penetrance. VUR may occur in up to 66% of the offspring of VUR patients [3], and the prevalence of VUR among siblings of index VUR patients is approximately 32% [4].

It has long been appreciated that there is an association between recurrent UTI, VUR and renal parenchymal scarring [5]. The traditional paradigm holds that once pathogenic bacteria establish infection in the bladder, the presence of VUR allows these bacteria to gain access to the upper tracts, invading the renal parenchyma and producing clinical acute pyelonephritis [6]. The resulting inflammatory cascade is presumed to result in tissue damage, fibrosis, and scarring in susceptible individuals.

In general, most management strategies for VUR have sought to address and defeat this process at various points along the pathogenic sequence. Medical management with antimicrobial prophylaxis seeks to maintain sterile urine, rendering the VUR itself relatively harmless, since there are no bacteria present to reach and invade the kidney. Antireflux surgery (ARS), in contrast, reconfigures the ureterovesical junction anatomy to block access to the upper tracts, so that any episodes of cystitis that do occur cannot progress to pyelonephritis.

However, this model has been called into question in recent years by data that challenges many of the assumptions of the VUR paradigm. Long-term studies show that renal scarring can occur in children without VUR, and that renal scarring is not common in children with even high degrees of reflux [7, 8]. Rushton et al. noted that new renal scars are formed less frequently after acute pyelonephritis in kidneys with VUR than those without VUR [9], and other studies have supported these findings to some extent [10]. End-stage renal disease and transplant registries maintained since the 1960's have not demonstrated the reduction in the proportion of cases attributable to VUR that one would expect, if the management strategies instituted since that time were having a significant impact on rates of renal scarring and renal insufficiency [7, 11].

As we will see below, it has been difficult to demonstrate that current management strategies for VUR result in measurably improved outcomes. Since these management strategies are based on assumptions about the pathophysiology of UTI, VUR, and renal scarring, if such assumptions are incorrect then it should not surprise us that our interventions seem to have little or no effect.

## 2. MEDICAL MANAGEMENT

The use of antimicrobials to reduce recurrent and/or chronic UTI's dates back to the 1940's and 50's, and is the mainstay of initial management in children diagnosed with VUR. Based on the perception that antimicrobial prophylaxis is safe, effective, and easily tolerated, generations of children with VUR have spent years undergoing this treatment while awaiting the spontaneous resolution of their VUR. The classic studies of Smellie et al. form much of the basis for prophylaxis as a management tool [2, 5, 12]. In their numerous series, the Smellie group made seminal observations regarding the associations between VUR, UTI, and negative renal outcomes including scarring and decreased renal growth, and developed hypotheses regarding the apparent benefits of antimicrobial prophylaxis in children with VUR. They noted that children on continuous antimicrobial prophylaxis seemed to have fewer recurrent UTI than those on intermittent antibiotics, that children who stopped antibiotics seemed to be prone to recurrence shortly thereafter, and that increasing number of infections was associated with increased risk of renal scarring. Although groundbreaking, these data were based on nonrandomized, retrospective reviews, and thus do not adequately control for confounding factors and bias.

As a consequence, antimicrobial prophylaxis lacks basic evidence of efficacy in prevention of either UTI or renal scarring. Three randomized controlled trials comparing antimicrobial prophylaxis with no treatment (surveillance only) have been reported [13–15], and one of these was published in conference proceedings only [14]. None of the trials found significant differences in rates of UTI or renal scarring in treatment versus nontreatment groups. In the most recent study [15], subjects were kept on antimicrobial prophylaxis or no treatment for 2 years, and then were followed off medication for an additional 2 years. There were no differences in UTI rates either at the 2-year or 4-year mark. A recent population-based study using administrative data in a group of 611 children with UTI (27% of whom had VUR) found that the use of antimicrobial prophylaxis was not associated with decrease in risk of recurrent UTI [16]. Although each of these studies has methodological problems, the failure of any of them to find any effect of antimicrobial prophylaxis in preventing UTI suggests that the effect, if any, is likely to be very small. This, in turn, suggests that large number of children need to be treated for any single child to experience the benefits of prophylaxis.

## 3. SURGICAL MANAGEMENT

Since the initial report of surgical correction of VUR by Hutch in 1952 [17], numerous techniques have been developed to accomplish the basic goals of ARS, that is, prevention of retrograde flow of urine into the ureter and kidney. In fact, many of the leading figures in the development of the specialty of pediatric urology made their names largely through their accomplishments in perfecting ARS techniques. Today, in expert hands, the success rate of straightforward ARS approaches 100%, such that some surgeons no longer bother with post-ARS cystography to confirm VUR resolution [18–20].

The extraordinary success of modern ARS might lead one to assume that there is little room left for technical innovation in this field. However, investigators have long sought a less invasive way to correct VUR. In 1981, the first injection technique was reported by Matouschek using polytetrafluoroethylene (PTFE; Teflon) paste [21]. Concern over migration of PTFE particles to distant body sites [22] limited the popularity of this bulking agent in the United States, but in 1995, a Swedish group reported development of a dextranomer copolymer/hyaluronic acid gel for use as an injectable bulking agent (DX/HA; Deflux) [23]. The FDA approved Deflux for correction of VUR in 2001, and since then its use has increased significantly in many parts of the US [24] , with reported VUR resolution rates of 68–89% [25–28].

To our knowledge, there have not been any prospective trials of surgical management compared with observation in children with VUR. Therefore, we simply do not know if ARS is superior to surveillance alone in prevention of UTI or renal scarring. Because active management of VUR (either with antibiotics or surgery) is considered standard of care, it is difficult to find patients who have truly been given no treatment for their VUR, even in a retrospective review.

## 4. COMPARISON OF SURGICAL VERSUS MEDICAL MANAGEMENT

Comparison of medical treatment with surgical treatment for VUR is challenging because the different studies have used various outcome measures, and even studies using similar outcome measures may be difficult to compare due to differing definitions of similar outcomes. Reported outcomes in many studies include postoperative incidence of any UTI, incidence of febrile UTI (presumed in most cases to be equivalent to pyelonephritis), and renal cortical abnormalities (scarring).

In a recent metaanalysis of clinical trials, Hodson et al. identified seven randomized controlled trials comparing surgical and medical management [29–35], and summarized their results [36]:

### 4.1. Any UTI

There was no difference in incidence of any UTI between treatment groups, with incidence of 29–42% in antibiotic only group and 25–40% in the surgical group [29–35]. Thus, surgical treatment of VUR does not seem to reduce the rate of UTI overall.

### 4.2. Febrile UTI

Reported in only 2 studies, this is the only outcome where significant differences in outcomes have been observed between treatments [31, 37]. The surgical group had significantly fewer febrile UTI's in short-term and long-term followup [32], with relative risk of febrile UTI during the first 5 years of 0.43 (95% CI: 0.27–0.70).

### 4.3. Renal scarring

In the five studies that assessed renal parenchymal abnormalities using IVP criteria [29–31, 33, 38], there were no significant differences noted between surgical and medical groups. The majority of these studies assessed renal abnormalities using IVP. In the two studies that reported DMSA renal scintigraphy [35, 39], there was no difference in either progression of existing scars or development of new scars.

### 4.4. Future directions

There is little strong evidence supporting the hypothesis that early detection and treatment of VUR is of any benefit, primarily because it has been so difficult to demonstrate any benefit from the available therapies. Perhaps the one firm conclusion we can draw from the literature described above is that, among children with VUR who have had breakthrough febrile UTI's while on antimicrobial prophylaxis, ARS is an appropriate therapy that can be expected to reduce the incidence of such febrile episodes. However, neither prophylaxis nor ARS can be reliably stated to reduce the risk of new or progressive renal scarring, although it is prevention of this outcome that is widely assumed to be the most important benefit of VUR treatment.

It is plausible that, while treatment of VUR may reduce the risk of negative outcomes in a small subset of VUR patients, the number needed to be treated (in order to realize those benefits); it may be so high as to make the intervention unjustified for the overall VUR population. For this reason, ongoing research into biomarkers that will indicate those at highest risk for recurrent infection and progressive renal damage is crucial; such biomarkers would allow us to narrow the field of candidates for medical or surgical treatment to those most likely to benefit, and allow the larger VUR population to escape the morbidity and bother associated with these treatments.

Finally, there has been much recent discussion about whether the availability of endoscopic ARS should alter the indications for ARS. Suggestions have begun to appear in the literature and at national meetings that endoscopic treatment should be utilized as initial therapy for patients diagnosed with VUR. Advocates argue that immediate endoscopic therapy is preferable to antimicrobial prophylaxis in children just diagnosed with VUR [40]. Current standards of care do not yet embrace such early treatment: Khoury and Bagli state in their textbook chapter that “the indications for correction of reflux should remain unchanged regardless” of technique [41]. Furthermore, the data shown above make it clear that immediate ARS (using any method) makes little sense: the only demonstrated benefit of ARS is the reduction in incidence of recurrent febrile UTI, and a majority of newly diagnosed patients with VUR (except those with high-grade VUR) will never experience a recurrent febrile UTI, regardless of treatment choice [13, 16]. Therefore, an algorithm that directs all newly diagnosed VUR patients into immediate surgical treatment (even if it is the “low morbidity” of endoscopic ARS) is destined to overtreat large numbers of children for whom there will not be measurable benefits.

Ongoing clinical studies will hopefully clarify some of the glaring shortcomings in our evidence base. The NIDDK-funded RIVUR study is a randomized trial of antimicrobial prophylaxis versus placebo in children with VUR and UTI [42]. Each subject is followed for 2 years during which incidence of UTI and renal scarring by DMSA criteria will be tracked. DMSA scans will be obtained at study entry, 1 year, and 2 years. Weaknesses of the study will include the broad range of subjects (intended to increase generalizability), including boys and girls, VUR Grade I-IV, ages 2 months to 5 years, and inclusion of trained and nontoilet trained children, with or without voiding dysfunction. Although the 2-year time frame is short, this large study (target sample *n* = 600) will provide us with superb data regarding risk of UTI and renal scarring in children with VUR in the short term, as well as demonstrate whether antimicrobial prophylaxis is effective in preventing either UTI or scarring. Other studies assessing the utility of ARS in various clinical scenarios are desperately needed. Until such studies are complete, clinicians who treat children with VUR will continue to rely on clinical judgment, experience, and intuition to manage their young patients.
